# The genetics of aniridia — simple things become complicated

**DOI:** 10.1007/s13353-017-0426-1

**Published:** 2018-02-19

**Authors:** Anna Wawrocka, Maciej R. Krawczynski

**Affiliations:** 1 Department of Medical Genetics, Poznan University of Medical Sciences, Rokietnicka 8, 60-806 Poznan, Poland; 2 Centers for Medical Genetics GENESIS, Poznan, Poland

**Keywords:** Aniridia, *PAX6*, 11p13 microdeletions, MLPA, High-resolution array-CGH

## Abstract

Aniridia is a rare, panocular disorder characterized by a variable degree of hypoplasia or the absence of iris tissue associated with additional ocular abnormalities. It is inherited in an autosomal dominant manner, with high penetrance and variable expression even within the same family. In most cases the disease is caused by haploinsufficiency truncating mutations in the *PAX6* gene; however, in up to 30% of aniridia patients, disease results from chromosomal rearrangements at the 11p13 region. The aim of this review is to present the clinical and genetic aspects of the disease. Furthermore, we present a molecular diagnostic strategy in the aniridia patients. Recent improvement in the genetic diagnostic approach will precisely diagnosis aniridia patients, which is essential especially for children with aniridia in order to determine the risk of developing a Wilms tumor or neurodevelopmental disorder. Finally, based on the previous studies we describe the current knowledge and latest research findings in the topic of pathogenesis of aniridia and possible future treatment.

## Introduction

**Fig. 1:**
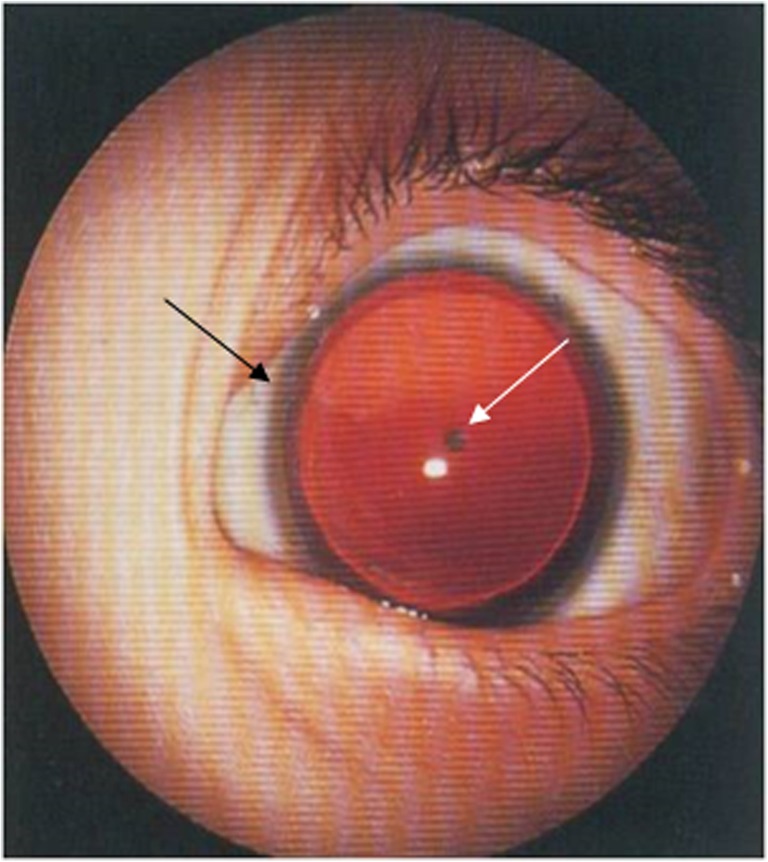
A typical phenotype of a complete aniridia with polar posterior cataract (white arrow) and peripheral iris remnants (black arrow)

Aniridia (OMIM 106210) is a congenital panocular disorder characterized by complete or partial iris hypoplasia presenting in early infancy. Clinical expression is highly variable between families and even in the same family. Foveal hypoplasia resulting in reduced visual acuity, as well as cataract, keratopathy, and glaucoma that sometimes develop in the second or third decade, contribute to visual morbidity (Fig. 1). Visual acuity is variable, it is usually 20/100-20/200, but in some affected individuals it can be better than 20/60 especially when nystagmus is not present (Hingorani et al. 2012). Refractive errors, strabismus and ptosis may also be present in the affected eye (Kokotas and Petersen 2010).

Some aniridia patients have been reported to present with non-ocular, sensory, and neurological abnormalities. Reduced olfaction seems to be the most common functional deficit (Sisodiya et al. 2001; Hingorani et al. 2012). Neurological abnormalities such as autism and intellectual disability have been described by several authors (Davis et al. 2008; Hu et al. 2015). MRI studies showed irregularities of the anterior commissure, anterior cingulate cortex, cerebellum, temporal and occipital lobes, corpus callosum, pineal gland and olfactory bulb (Sisodiya et al. 2001; Mitchell et al. 2003; Bamiou et al. 2007). Abnormal interhemispheric transfer results in central auditory processing deficits, what can cause hearing difficulties (Bamiou et al. 2007).

Aniridia is an autosomal dominant condition with an incidence of approximately one in 40,000-100,000 live births worldwide (Kokotas and Petersen 2010; Hingorani et al. 2012). About two-thirds of aniridia cases are familial with nearly complete penetrance, the remaining one-third are sporadic and result from de novo mutations (Hingorani et al. 2012). Aniridia can occur either as an isolated malformation or as a part of a syndrome. The best known syndromic form is WAGR syndrome (OMIM 194072) — a contiguous genes deletion syndrome of the 11p13 region (encompassing the *PAX6* and *WT1* genes). It is characterized by Wilms tumor, aniridia, genitourinary anomalies, and intellectual disability. Patients have 50-70% risk to develop Wilms tumor, which is the most common form of kidney cancer in children. Approximately 30% of patients with the sporadic form of aniridia suffer from this syndrome (Fischbach et al. 2005). Another known form of aniridia is Gillespie syndrome (OMIM 206700) — a rare disorder characterized by non-progressive cerebellar ataxia, intellectual disability, and iris hypoplasia. Gillespie syndrome is estimated to account for about 2% of aniridia-like cases (Gillespie 1965; Ansari et al. 2016). The *PAX6* gene mutations were identified in only two individuals described as Gillespie syndrome but with atypical features like corectopia and ptosis (Ticho et al. 2006). In 2016, Gerber et al. identified mutations in the *ITPR1* gene in patients affected with Gillespie syndrome (Gerber et al. 2016); however, in most cases the genetic cause of the disease remains unclear.

## Structure and function of the *PAX6* gene and its protein

The *PAX6* gene (OMIM 607108) was the first homeobox gene discovered to play a crucial role in eye development (Glaser et al. 1992). In 1991 it was identified by positional cloning as a causative gene for congenital aniridia (Ton et al. 1991; Glaser et al. 1992).

**Fig. 2:**
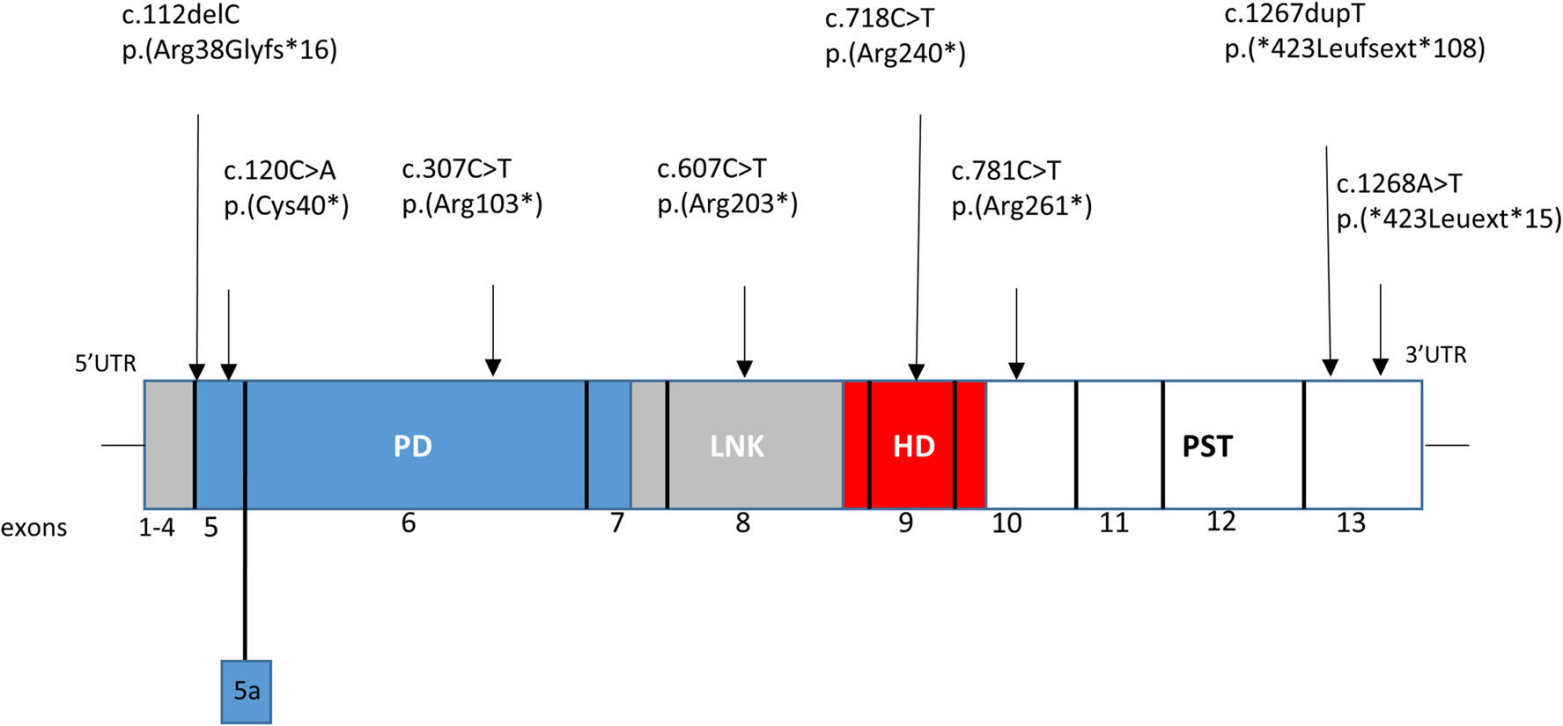
The *PAX6* cDNA, horizontal rectangles represent PAX6 protein domains including: PD-paired domain, LNK-linker region, HD-homeodomain, PST-proline/serine/threonine rich region. Exon 5a is an alternatively spliced exon in the PD domain. The most frequent mutations in the *PAX6* gene described in LOVD database are presented above the diagram

The *PAX6* gene is located on chromosome 11p13, contains 14 exons including an alternatively spliced exon 5a. It is abundantly expressed in the forebrain, early eye structures, ventral spinal cord, and endocrine pancreas (Lauderdale et al. 2000). The PAX6 protein as a transcriptional factor plays a crucial role in embryonic development and organogenesis, including neuro- and oculogenesis (Lang et al. 2007; Kokotas and Petersen 2010). The protein contains two functional domains: paired domain (PD) and homeodomain (HD), separated by the proline/serine/threonine-rich transactivation domain (PST). The paired domain comprises two structurally distinct DNA-binding subdomains: the N-terminal subdomain (NTS) and the C-terminal subdomain (CTS) (Kokotas and Petersen 2010) (Fig. 2). The *PAX6* gene produces two alternatively spliced isoforms, with a distinct structure of the PD domain: a 422 amino acid and an alternatively spliced 436 amino acid PAX6 protein (including exon 5a). The insertion, into the NTS subdomain, of 14 additional amino acids encoded by exon 5a abolishes the DNA-binding activity of the NTS and unmasks the DNA-binding ability of the CTS. Thus, exon 5a appears to function as a molecular switch that specifies target genes (Azuma et al. 1999).

## Heterozygous *PAX6* gene point mutations

To date, more than 400 heterozygous variants of the *PAX6* gene have been identified and recorded in the Human *PAX6* Allelic Variant Database (Leiden Open Variation Database, LOVD). Of all intragenic point mutations in the *PAX6* gene, 94% result in premature termination codon (PTC), C-terminal extensions (CTE) or amino-acid substitutions (missense mutations) (Hingorani et al. 2012). Most of the reported *PAX6* gene heterozygous mutations including nonsense and splicing mutations, insertions, and deletions, introduce premature termination codons into the PAX6 open reading frame, resulting in haploinsufficiency. The level of the protein expression from a single functional allele is not sufficient to produce biologically active PAX6 protein (50% reduction in overall activity) (Kokotas and Petersen 2010). The dosage-dependance of the *PAX6* gene was supported by studies in naturally occuring rodent models with heterozygous *Pax6* mutations (Matsuo et al. 1993; Kokotas and Petersen 2010; Cvekl and Callaerts 2017). Heterozygous mutations in the *Pax6* gene cause mouse smalleye (Sey) phenotype demonstrating dosage sensitivity (Hill et al. 1991).

Truncating mutations activate the nonsense-mediated decay (NMD) process, by which mRNA containing premature termination codons are degraded before they produce truncated proteins (Baker and Parker 2004).

C-terminal extension mutations (dominant negative mutations) in the *PAX6* gene result from frameshift or point mutations that change the stop codon of the C-terminus to cause run-on translation into 3’UTR. Nonsense-mediated decay is not predicted for CTE transcripts and an extended abnormal protein is produced (Tzoulaki et al. 2005). This type of mutation is usually associated with severe phenotype with pronounced iris hypoplasia and profound visual impairment (Aggarwal et al. 2011).

Single amino acid substitutions (missense mutations) produce a full-length protein that may fold and function abnormally, causing phenotypes ranging from mild iris defects to more severe including optic nerve malformations, Peters anomaly and microphthalmia (Hanson et al. 1994; Grønskov et al. 2001; Azuma et al. 2003).

Heterozygous *PAX6* gene mutations account for approximately two-thirds of all aniridia cases (Robinson et al. 2008). The most frequent mutations in the *PAX6* gene are present in Fig. 2.

## Compound heterozygous *PAX6* gene mutations

Families of aniridic parents with heterozygous *PAX6* gene mutations were reported in the literature. Affected parents have a 25% risk that they would give birth to a child with both alleles having *PAX6* gene mutations. Compound heterozygosity in the *PAX6* gene was thought to be lethal, resulting in severe malformations of the fetuses. However, in 1994, Glaser et al. reported the first patient with compound heterozygous *PAX6* gene mutations. Two nonsense mutations inherited from the aniridic parents were identified in a newborn with anophthalmia, severe craniofacial, and central nervous system defects. The pattern of malformations was similar to that observed in homozygous Sey mouse (Glaser et al. 1994). Compound heterozygosity was also reported in two fetuses whose parents were affected with aniridia. Both fetuses showed severe brain malformations with increased germinal proliferation, gross disturbances of migration, and organization of the central nervous system (Schmidt-Sidor et al. 2009).

Finally, Solomon et al. reported a patient, who suffered from trisomy 21 and additionally was a compound heterozygote for one missense and one nonsense mutations in the *PAX6* gene, inherited from the parents. The presence of both mutations resulted in severe ophthalmological, neurological, and endocrinological disturbances. This four year old patient suffers from microphthalmia, neonatal diabetes mellitus, hypopituitarism, and a complex structural brain anomaly. This was the first reported patient who survived the neonatal period (Solomon et al. 2009).

## 11p13 microdeletions

In 30% or even in higher proportion of aniridia patients (Wawrocka et al. 2013), the disease results from genomic rearrangements at the 11p13 region including *PAX6* whole-gene deletions, microdeletions affecting only the 3′ regulatory enhancers (excluding the *PAX6* gene) or contiguous genes deletions of the *PAX6* and other neighboring genes, especially *WT1* (WAGR syndrome) (Crolla and van Heyningen 2002; Robinson et al. 2008).

## Microdeletions comprising the *PAX6* gene and the adjacent genes

Microdeletions encompassing the *PAX6* and the neighboring genes have been described by several authors (Fantes et al. 1992; Crolla and van Heyningen 2002; Hu et al. 2015). Identified deletions were noted even though the *PAX6* gene usually harbors doublecortin domain-containing protein 1 (*DCDC1*), elongation factor protein (*ELP4*), brain-derived neurotrophic factor (*BDNF*), beta polypeptide of follicle-stimulating hormone (*FSHB*), reticulocalbin 1 (*RCN1*), partial IMP1 inner-mitochondrial membrane (*IMMP1L*) (Wawrocka et al. 2013; Hu et al. 2015) and *WT1* gene (Crolla and van Heyningen 2002). Sporadic aniridia cases with a deletion of both *PAX6* and *WT1* genes have been determined in a number of studies (Grønskov et al. 2001; Muto et al. 2002). Deletions including the *PAX6* gene and other neighboring genes do not change the clinical expression of the disease in relation to mutation limited only to *PAX6*. However, for larger deletions encompassing *PAX6* and *WT1* genes, separated by 700 kb, patients with aniridia represent a more severe phenotype — WAGR syndrome with intellectual disability and high risk of Wilm’s tumor development.

## Microdeletions excluding the *PAX6* gene — position effect

In 1995, Fantes et al. described two aniridia families with genomic rearrangements at the 11p13 region, that did not include the *PAX6* gene sequence and proposed that the causative mechanism of the disease in these families is a “position effect” (Fantes et al. 1995). Thereafter, submicroscopic rearrangements that do not disrupt the *PAX6* gene sequence in the aniridia patients were described by several authors. Lauderdale et al. presented two de novo deletions of 11p13, located more than 11 kb downstream of the *PAX6* gene. Using human–mouse cell hybrids they showed that the *PAX6* gene expression is restricted to normal allele, unambiguously indicating that 3′ regulatory elements are essential for the *PAX6* gene expression (Lauderdale et al. 2000). Davis et al. identified in a patient with aniridia, autism and intellectual disability a 1.3 Mb deletion, situated approximately 35 kb downstream of the *PAX6* gene, comprising the *ELP4*, *DPH4*, *DCDC1*, *DCDC5*, *MPPED2*, and *IMMP1L* genes (Davis et al. 2008). Genomic rearrangements downstream of the *PAX6* gene identified so far in aniridia patients are summarized in Table 1.

**Table 1:** Deletions in the 11p13 region, downstream of the *PAX6* gene reported previously in the literature

Deleted genes (whole or partially)	Distance from the 3′ of *PAX6*	Genomic coordinates (hg19)	Size of deletion	Phenotype	References
**Aniridia or aniridia and other malformations**					
*ELP4, IMMP1L, DNAJC24, DCDC1, DCDC5*	22.1 kb	ND	975 kb	aniridia	(Lauderdale et al. 2000)
*ELP4, IMMP1L, DNAJC24, DCDC1, DCDC5*	11.6 kb	ND	1105 kb	aniridia	
*ELP4*	5 kb	ND	ND	aniridia, cataracts, glaucoma	(D’Elia et al. 2007)
*ELP4, IMMP1L, DNAJC24, DCDC1, DCDC5, MPPED2*	35 kb	chr11:30,448,178-31,802,357	1.3 Mb	aniridia, autism, mental retardation	(Davis et al. 2008)
*ELP4, IMMP1L, DNAJC24, DCDC1*	140 kb	chr11:31,260,340-31,666,340	406 kb	aniridia	(Bayrakli et al. 2009)
*ELP4, IMMP1L, DNAJC24, DCDC1*	123 kb	chr11:31,117,827-31,683,687	566 kb	aniridia	(Cheng et al. 2011)
*ELP4, IMMP1L, DNAJC24, DCDC1*	1 kb	chr11:31,280,628-31,805,329	525 kb	aniridia	(Zhang et al. 2011)
*ELP4, IMMP1L, DNAJC24, DCDC1*	85 kb	chr11:31,122,161-31,721,030	599-652 kb	partial aniridia	(Wawrocka et al. 2012)
*ELP4*	8 kb	chr11:31,706,160-31,755,245 bp	49 kb	aniridia, developmental delay, a submucous cleft palate, ventriculoseptal defect	Simoni et al. 2012
*ELP4, IMMP1L, DNAJC24, DCDC1*	96 kb	chr11:31,118,027-31,710,576	593 kb	Rieger anomaly, aniridia	(Addis et al. 2015)
*ELP4, IMMP1L, DNAJC24, DCDC1*	31 kb	chr11:31,172,410-31,775,457	603 kb	aniridia	
*ELP4*	23 kb	chr11:31,605,859-31,783,590	178 kb	partial aniridia	
*ELP4, IMMP1L, DNAJC24, DCDC1, DCDC5*	108 kb	chr11:30,918,066-31,698,257	780 kb	aniridia	(Ansari et al. 2016)
*ELP4, IMMP1L, DNAJC24, DCDC1, DCDC5*	59 kb	chr11:31,010,424-31,747,424	737 kb	aniridia	
*ELP4, IMMP1L, DNAJC24, DCDC1*	113 kb	chr11:31,152,003-31,693,266	541 kb	aniridia	
*ELP4, IMMP1L, DNAJC24*	54 kb	chr11:31,422,424-31,751,424	329 kb	aniridia	
*ELP4, IMMP1L, DNAJC24, DCDC1*	11 kb	chr11:31,277,819-31,795,239	517 kb	partial aniridia, ataxia, developmental delay	
*ELP4, IMMP1L, DNAJC24, DCDC1*	91 kb	chr11:31,147,306-31,714,853	567 kb	aniridia	(Blanco-Kelly et al. 2017)
*ELP4, IMMP1L, DNAJC24, DCDC1*	108 kb	chr11:31,186,493-31,698,208	512 kb	aniridia	
*ELP4, IMMP1L, DNAJC24, DCDC1*	101 kb	chr11:31,083,877-31,704,548	620 kb	aniridia	
**Ocular malformations and/or neurodevelopmental disorders without aniridia**					
*ELP4, IMMP1L, DNAJC24, DCDC1, DCDC5*	114 kb	chr11:31,010,914-31,692,238	681 kb	ocular coloboma	(Guo et al. 2013)
*ELP4, IMMP1L, DNAJC24*	191 kb	chr11:31,452,082-31,615,319	163 kb	intellectual disability, speech abnormalities, autistic behaviors	(Balay et al. 2016)
*ELP4, IMMP1L, DNAJC24, DCDC1, DCDC5*	242 kb	chr11:30,991,456-31,564,708	573 kb	focal epilepsy with cortical dysplasia, mild developmental delay, ADHD, neurinomas, squint, ptosis, fine motor dyspraxia	
*ELP4, IMMP1L*	260 kb	chr11:31,495,260-31,546,276	51 kb	cognitive delay, speech and language disorder, reading and spelling disorder, ASD, epilepsy	
*ELP4*	181 kb	chr11:31,561,220-31,625,448	64 kb	speech and language delay	
*ELP4*	132 kb	chr11:31,573,422-31,674,789	101 kb	developmental delay, microcephaly	
*ELP4*	164 kb	chr11:31,584,329-31,642,325	58 kb	language disorder, behavioral problems	
*ELP4*	174 kb	chr11:31,601,768-31,632,347	31 kb	developmental delay, hypotonia, ventriculomegaly	(Addis et al. 2015)
*ELP4*	84 kb	chr11:31,691,270-31,722,740	31 kb	developmental delay, speech and language disorder, microcephaly, mild cognitive delay, motor skills development disorder	
*ELP4*	59 kb	chr11:31,705,076-31,747,631	43 kb	autism, learning difficulties	
*ELP4*	20 kb	chr11:31,760,904-31,786,914	26 kb	moderate developmental delay, autism	
*ELP4*	4 kb	chr11:31,597,322-31,802,120	205 kb	severe intellectual disability, muscle hypotrophy, severe dysphagia, craniofacial abnormalities	
*ELP4*	31 kb	chr11:31,605,859-31,775,457	170 kb	developmental delay, behavioral problems, pervasive developmental disorder	
*ELP4*	31 kb	chr11:31,625,389-31,775,457	150 kb	behavioral and speech problems, mild mental retardation	
*ELP4, IMMP1L*	151 kb	chr11:31,460,506-31,655,108	195 kb	autism	
*ELP4, IMMP1L*	199 kb	chr11:31,488,890-31,607,986	119 kb	autism	(Addis et al. 2015)
*ELP4, IMMP1L*	157 kb	chr11:31,518,924-31,649,475	131 kb	autism, language delay	
*ELP4*	76 kb	chr11:31,576,768-31,653,568	77 kb	autism, coordination problems	
*ELP4*	112 kb	chr11:31,652,219-31,764,393	112 kb	autism, language delay, mild developmental delay, motor delay	

ND – no data, ASD – autism spectrum disorder, ADHD – attention deficit hyperactivity disorder

Cis-regulatory elements (CREs) such as promoters, enhancers, insulators, and boundary elements play an important role in the regulation of gene expression, they determine the time, place, and the level of the expression of their target genes. Cis-regulatory elements can spread hundreds of kilobases upstream or downstream from the transcription start site of the genes. Human genetic studies using YAC transgenic mouse, DNaseI hypersensitivity mapping and reporter transgenic assays revealed a presence of SIMO enhancer located in the 3′- distal region, 124 kb from the PAX6 polyadenylation site, a retina-specific enhancer located within a fragment containing HS2 and HS3 elements and a lens-specific enhancer. These sequences located within introns of the adjacent *ELP4* gene are PAX6-specific long range control elements (DRR – downstream regulatory region) (Kleinjan et al. 2001, 2002). DNaseI hypersensitivity and ultraconservation analysis carried out by McBride et al. allowed identification of new long range enhancers HS5 and HS6 at the Pax6 cis-regulatory region. HS5 plays a role in maintaining the *PAX6* gene expression in the optic cup and diencephalon, while HS6 drives the *PAX6* gene expression in the developing eye and the precerebellar neuron-epithelium of the hindbrain in a time specific manner during development (McBride et al. 2011). Deletion of the DRR region (including the retina enhancer in the HS2 and HS3 fragment) in mouse abolished Pax6 expression in the retina, iris, and ciliary body indicating that the presence of the DRR region is indispensable in this structure (Kleinjan et al. 2006). Additional cis-regulatory elements may be present in the DRR region, but have not been discovered yet.

Deletion of the SIMO enhancer in mouse did not completely abolish the *Pax6* gene expression in the lens due to the presence of additional enhancers regulating its expression in this tissue (Kleinjan et al. 2006). However, Bhatia et al. demonstrated using the BAC-transgenic approach in zebrafish and mouse reporter that continued tissue-specific expression from the Pax6 promoters is critically dependent on the presence of the SIMO enhancer. The point mutation that disrupts the autoregulatory PAX6 binding site within SIMO causes loss of enhancer activity and abolishes lens and late retinal expression in transgenic mouse. The identified mutation of an autoregulatory binding site revealed a distinct regulatory mechanism by disruption of a positive feedback loop critical for gene expression during development (Bhatia et al. 2013).

The previous studies showed that genomic sequences located in a region of approximately 120 kb 3′ to the transcription start site are important for transcriptional activation of the *PAX6* gene. Recent studies performed by Ansari et al. in a cohort of aniridia and Gillespie syndrome patients negative for the *PAX6* gene mutations suggest a new “critical region” of 244 kb that includes cis-regulatory elements, essential for PAX6 transcriptional activation. This region spans a fragment of the *DNAJC24*, *IMMP1L* genes, and introns 1-7 of the *ELP4* gene (chr11:31,379,000 (hg18) and 31,622,916 (hg18)) (Ansari et al. 2016).

**Fig. 3:**
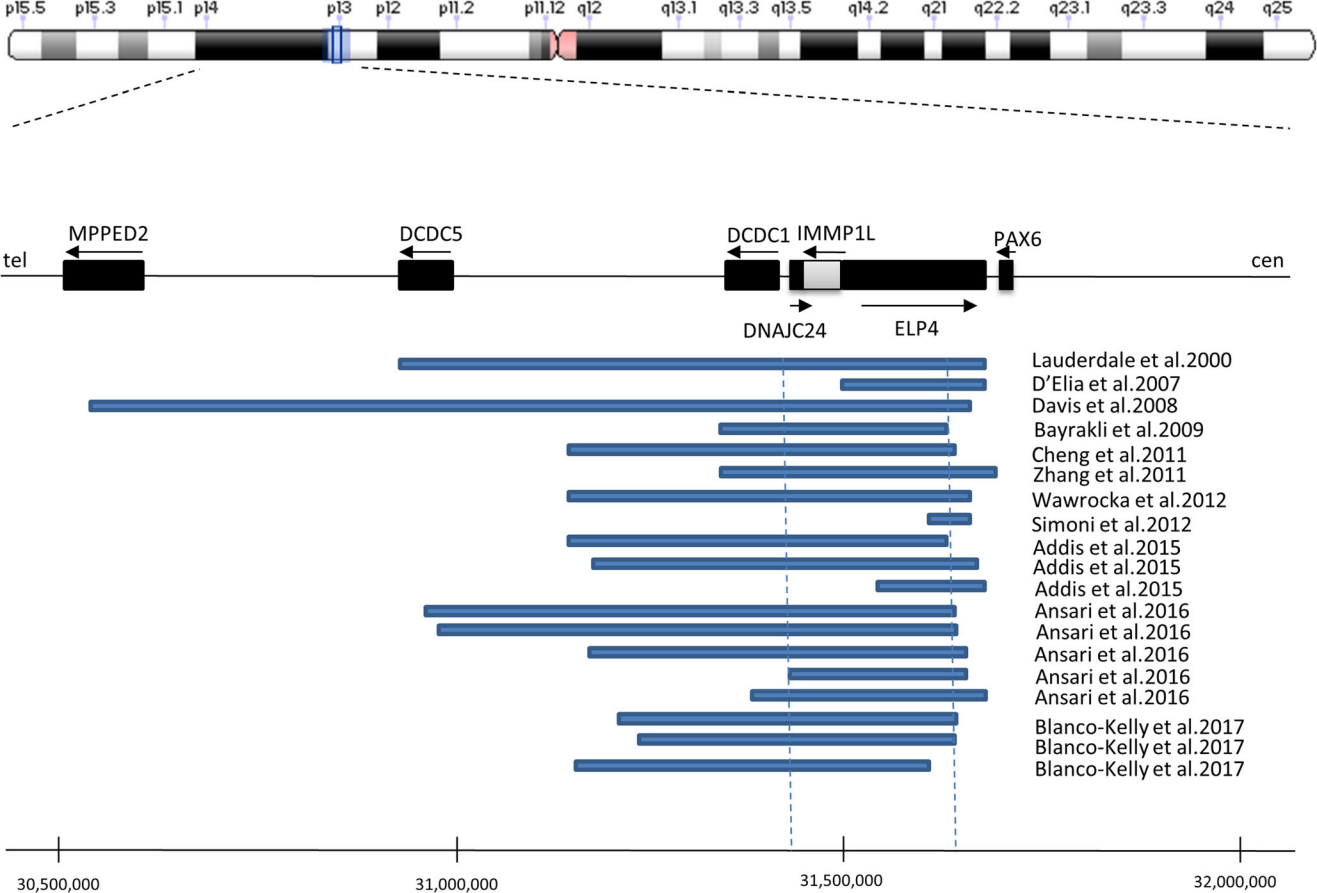
Schematic diagram of the previously reported microdeletions in the 11p13 region, downstream of the *PAX6* gene in the aniridia patients. Horizontal blue blocks represent deletions that have been identified in the aniridia patients. The vertical blue dashed lines indicate the “critical region” required for the *PAX6* gene transcription suggested by Ansari et al. 2016

Microdeletions including or disrupting the *ELP4* gene were also shown in patients with neurodevelopmental disorders including autism spectrum, speech/language disorders, epilepsy, and developmental delay. Microdeletions identified by Addis et al. do not involve the entire critical region. Only in two patients with aniridia, deletions downstream of the *PAX6* gene including the *ELP4* gene affecting the entire critical region were identified (Fig. 3). Therefore, deletions including the critical region cause aniridia or other ocular malformation and may also predispose to neurodevelopmental disorders (Addis et al. 2015).

## *PAX6* gene mutations and 11p13 microdeletions in non aniridia patients

Although most mutations in the *PAX6* gene are responsible for aniridia, some mutations can be connected to some other ocular disorders including: microphthalmia, microcornea, foveal hypoplasia, ocular coloboma, keratitis, congenital cataract, Gillespie syndrome, Peters anomaly, and morning glory disc anomaly (Mirzayans et al. 1995; van Heyningen and Williamson 2002; Azuma et al. 2003; Tzoulaki et al. 2005; Wang et al. 2012). Aniridia is predominantly associated with truncating mutations in the *PAX6* gene, while missense mutations usually lead to non-aniridia phenotypes (Tzoulaki et al. 2005).

Genomic rearrangements at 11p13 region, have also been reported in non aniridia patients, suggesting that clinical manifestation may vary depending on the location of the breakpoints and genes involoved. The 681 kb deletion downstream of the *PAX6* gene including the *DCDC5, DCDC1, DNAJC24, IMMP1L* genes and a part of the *ELP4* gene has been discovered in a patient with ocular coloboma (Guo et al. 2013). A familial pericentric inversion of chromosome 11 associated with submicroscopic interstitial deletion of 11p13 and duplication of 11q22.3 was presented by Balay et al. The 11p13 deletion of 163 kb included the *IMMP1L* gene and part of the *DNAJC24* and *ELP4* genes. The patient’s phenotype was characterized by intellectual disability, speech abnormalities, and autistic behaviors; however, nobody in this family presented aniridia or other eye anomalies (Balay et al. 2016).

## Perspectives of the gene therapy

Identification of the *PAX6* gene transcription network, that means genes directly regulated by the *PAX6* gene, is essential in explaining tissue sensitivity to PAX6 dosage. Xia Wang et al., using laser capture microdissection in mouse, chromatin immunoprecipitation, promoter-reporter assays, and immunochemistry methods, demonstrated for the first time that the *Bmp4*, *Tgfβ2*, and *Foxc1* genes are direct downstream targets of Pax6 in developing iris and ciliary body (Wang et al. 2017b). In some patients with either no *PAX6* gene intragenic mutations nor 11p13 genomic rearrangements, mutations in the Pax6 downstream targets are the cause of aniridia. Furthermore, the previous study performed by Gregory-Evans et al. demonstrated that by after birth manipulation of Pax6 dosage using nonsense suppression, it is possible to prevent the development of aniridic phenotype in mouse. It suggests that downstream targets are also active after birth. These studies gives an opportunity for future treatment of aniridia using Pax6 targets (Gregory-Evans et al. 2014).

A nonsense suppression therapy is a promising therapeutic approach that has been introduced and successfully used in an animal model. In recent studies, Xia Wang et al. used a nonsense suppression to manipulate Pax6 dosage at different stages during eye development of the small eye mouse model of aniridia. They performed in vivo tests to study effects in the developing retina, cornea, and lens, using Ataluren — a compound with nonsense suppression activity, which suppresses the abnormal nonsense codon in the mRNA template. Eye malformations responded to delivered after birth nonsense supression in a manner dependent on the Pax6 dose and specific time. The obtained results seem to be very promising and give hope for aniridia patients with nonsense mutations. Nonsense mutation supression strategy could be significant for the patients if the drug could be targeted to the affected tissue at the appropriate time (Wang et al. 2017a).

This findings seems to be very important regarding the possible therapeutic startegies for patients with aniridia.

## Summary

In many studies it was proven that aniridia is a typical autosomal dominant disorder resulting from inactivation of one allele of the *PAX6* gene, predominantly through intragenic mutations leading to premature termination of the protein. Due to the fast development of the molecular diagnostic methods the genetic background of aniridia became much more complex. Chromosomal abnormalities at chromosome 11p13, including the *PAX6* gene deletions, contiguous genes deletions of the *PAX6* and other neighboring genes, as well as microdeletions affecting only the 3′ regulatory enhancers, have also been reported in association with both sporadic and familial cases of aniridia. The unusual and wide spectrum of molecular defects observed in the aniridia patients makes this disease a model monogenic disorder in terms of its molecular background.

Due to clinical and allelic heterogeneity, as well as the great variability of the genetic mechanisms involved in aniridia, molecular analysis of the patients is performed by combining different molecular approaches including Sanger sequencing, multiplex ligation-dependent probe amplification (MLPA), and an array based comparative genomic hybridization (aCGH). Although the intragenic *PAX6* gene mutations are more common than microdeletions, in newborns affected with aniridia the analysis toward deletions is recommended first, due to the clinical importance of early WAGR syndrome identification. At the same time this strategy gives an opportunity to detect genomic rearrangements at 11p13 in approximately 30% of aniridia patients. High resolution aCGH, and to some degree also commercially available MLPA set, allow for detection of small intragenic copy number variations (CNVs) at the resolution of single exons (Morice-Picard et al. 2014). Recently, Blanco-Kelly et al. developed a customized single locus aCGH for aniridia called WAGR-array with a high resolution CNV detection and precision of identified breakpoints (Blanco-Kelly et al. 2017). Precise mapping of the breakpoints is very important in accurate molecular diagnosis of children with aniridia in order to determine not only the risk of developing Wilms tumor but also neurodevelopmental disorder (deletion of the *ELP4* gene). In situations when MLPA, aCGH, and the *PAX6* gene sequencing do not identify any cause of the disease, a whole exome (WES) or whole genome sequencing (WGS) could be used for identification of novel (e.g., deep intronic) mutations in the *PAX6* gene or novel genes involved in pathogenesis of aniridia.

In our studies, despite using all the above mentioned techniques, there is still a proportion of aniridia patients that remain unsolved. Our patients do not have the *PAX6* gene mutations, nor genomic rearrangements that involve the *PAX6* gene or its regulatory elements. It suggests that mutations in the other genes, yet undiscovered genetic loci or unknown mechanisms disrupting the *PAX6* gene function could be the cause of aniridia. We await descriptions of both genocopies and possible recessive mechanisms of aniridia that are already suggested by the pedigree analysis.
